# Global health competencies and approaches in medical education: a literature review

**DOI:** 10.1186/1472-6920-10-94

**Published:** 2010-12-22

**Authors:** Robert Battat, Gillian Seidman, Nicholas Chadi, Mohammed Y Chanda, Jessica Nehme, Jennifer Hulme, Annie Li, Nazlie Faridi, Timothy F Brewer

**Affiliations:** 1 Faculty of Medicine, McGill University, Montreal, Canada

## Abstract

**Background:**

Physicians today are increasingly faced with healthcare challenges that require an understanding of global health trends and practices, yet little is known about what constitutes appropriate global health training.

**Methods:**

A literature review was undertaken to identify competencies and educational approaches for teaching global health in medical schools.

**Results:**

Using a pre-defined search strategy, 32 articles were identified; 11 articles describing 15 global health competencies for undergraduate medical training were found. The most frequently mentioned competencies included an understanding of: the global burden of disease, travel medicine, healthcare disparities between countries, immigrant health, primary care within diverse cultural settings and skills to better interface with different populations, cultures and healthcare systems. However, no consensus on global health competencies for medical students was apparent. Didactics and experiential learning were the most common educational methods used, mentioned in 12 and 13 articles respectively. Of the 11 articles discussing competencies, 8 linked competencies directly to educational approaches.

**Conclusions:**

This review highlights the imperative to document global health educational competencies and approaches used in medical schools and the need to facilitate greater consensus amongst medical educators on appropriate global health training for future physicians.

## Background

Health issues are increasingly transnational and in recent years the concept of global health has emerged to address these issues. Global health is the study and practice of improving health and health equity for all people worldwide through international and interdisciplinary collaboration [1]. Factors such as increasing international travel, the globalization of food supplies and commerce and the occurrence of multinational epidemics including the 2009 Influenza A pandemic have heightened awareness of global health issues. This awareness has influenced health practices and medical education locally and globally. Internationally, large-scale multinational public health programs such as the UN Millennium Development Goals, the Global Fund and the US President's Emergency Plan for AIDS relief have been created and funded with billions of dollars [2]. More locally, medical schools increasingly are offering international elective opportunities; almost one-third of recently graduated US and Canadian medical students participated in a global health experience [3]. However, despite growing interest in and the importance of global health, there exists little agreement on what constitutes appropriate global health training for medical students [4].

It has been argued that all medical students should have some exposure to global health issues, and groups are addressing this perceived gap in medical education by proposing global health competencies for undergraduate medical education [2,5,6]. In order to develop initial guidance in this area, this study reviewed existing literature to identify competencies and educational approaches recommended for teaching global health components in medical curricula. Using this information, a consensus was sought in order to further solidify this conceptual framework.

## Methods

### Data Sources and Searches

Relevant articles on global health competencies and teaching approaches were identified by applying similar search strategies to two databases, Ovid MEDLINE^® ^and Web of Science. Also, previously identified articles were obtained from the McGill Global Health Programs files. The Ovid MEDLINE^® ^search terms "world health" and "international educational exchange" were combined using the Boolean operator "OR" for the publication years 1996 to the 4^th ^week of January 2009. The initial search used the terms "global health" and "international health"; however, these terms mapped to the subject heading "world health" in the Ovid MEDLINE^® ^database. This procedure was repeated using the search terms "education, medical" and "education, medical, undergraduate", which was then cross-referenced with the search term "competencies". The resulting "education, medical"/"curriculum" set was combined with the resulting "world health"/"international educational exchange" set using the Boolean operator "AND". The results of the search were limited to humans and English. The Web of Science search cross-referenced the terms "medical education", "curriculum" and "global health" as topics for the publication years "all years" i.e. from 1900-1914 to January 2009. A research team comprised of all authors in this study, as well as the Liaison Librarian in the Life Sciences Library at McGill University, agreed upon these terms with the aim of avoiding researcher bias when selecting the articles. References from retrieved articles were reviewed to identify additional applicable publications.

### Study Selection

Titles and abstracts of articles obtained from database searches were reviewed to identify those describing competencies or educational approaches currently used in global health components of medical school curricula. Articles not pertaining to contemporary global health medical educational practices or competencies were not further considered.

### Data Extraction and Synthesis

Information relating to competencies and educational approaches was extracted from the retained articles. Information discussing the theoretical knowledge or practical skills authors believed medical students needed to obtain was categorized as a competency. Descriptions of specific programs or teaching methods were categorized as educational approaches.

## Results

**Figure 1:**
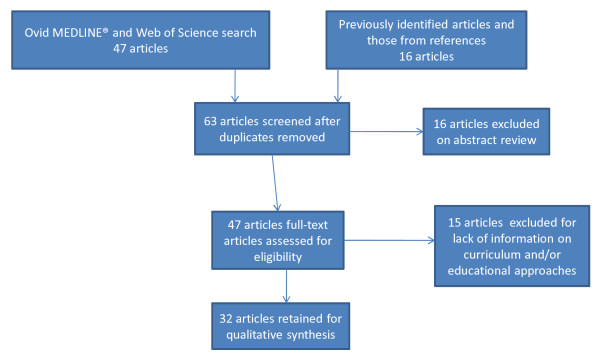
**Search strategy for retrieving literature on global health competencies and educational approaches**.

The results of the search strategy on global health competencies and educational approaches are summarized in Figure 1. The Ovid database search strategy yielded forty-five articles. The Web of Science search yielded two additional articles not found in the Ovid database[7,8]. Twelve more articles were found through reviewing references from retrieved articles[6,9-19]. Four other articles were obtained from the McGill Global Health Programs files[20-23]. Combining all of the search efforts and removing duplications, 63 articles were available for consideration. After reviewing titles and abstracts, 47 were retained for further consideration. Following a full review of the remaining articles, 32 articles were felt to contain relevant information and were included in the review.

The percentage of articles recommending a particular competency topic, the type of competency and the suggested method of implementation are summarized in Table 1. Fifteen unique global health competencies for training medical students from 11 articles (34.4%) were identified in the literature [7,10,17-19,24-29]. Most competencies were concerned with increasing medical students' knowledge, though some addressed physician behaviour, physical examination abilities and other clinical skills. Competencies mentioned in more than one article included: an understanding of the global burden of disease; travel medicine; healthcare disparities between countries; immigrant health; primary care within diverse cultural settings; and skills to better interface with different populations, cultures and healthcare systems. All other competencies were mentioned only by a single article.

**Table 1:** Global Health Competencies

Competency	Competency Type	%^a^	Methods of Implementation^b^
**Skills to better interface with different populations, cultures and healthcare systems **[18,19,26,28,29]	Knowledge/Behaviour	15.6	• Achieve meaningful community activities: experience working with at least 1 refugee family at a shelter for newly arriving refugee. • Lunch time seminars from faculty member or guest speakers with experience in medicine abroad • International health elective in fourth year • Workshop in cross-cultural communication: sensitize students to cultural differences that influence communication, teach how to use translators by interviewing standardized patients portraying cross-cultural scenarios.

**An understanding of immigrant health**[7,25,29]	Knowledge/Behaviour	9.4	• Internet-based training module focusing on refugees' experiences • Self-assessment quiz focused on global and refugee health • Cultural sensitivity workshop provided by medical faculty with expertise in refugee health.

**Primary care within diverse cultural settings**[24,26,29]	Physical exam/Clinical skills	9.4	• 4-8 weeks resident rotations • Second or third year internal medicine residents; 3 clinical rotations in the affiliated medical center of the host country • Community activities: working with refugee families at a refugee shelter

**Understand healthcare disparities between countries**[17,26]	Knowledge	6.3	• Work with patients and healthcare professionals in international locations

**An understanding of the burden of global disease**[7,25]	Knowledge	6.3	• Teaching about world health reports and Disability-Adjusted Life Years (DALYs)

**An understanding of travel medicine**[7,25]	Knowledge	6.3	Not described

**Develop a sense of social responsibility**[24]	Knowledge/Behaviour	3.1	• 4-8 weeks resident rotations • Second or third year residents in the internal medicine training programs. • Emphasis: clinical rotations in the affiliated medical center of the host country.

**Appreciate contrasts in healthcare delivery systems and expectations**[17]	Knowledge	3.1	• Work with patients and healthcare professionals in international locations.

**Humanism**[10]	Knowledge/Behaviour	3.1	Not described

**Scientific and societal consequences of global change**[27]	Knowledge	3.1	Not described

**Evolving global governance issues**[27]	Knowledge	3.1	Not described

**Cost of global environmental change**[27]	Knowledge	3.1	Not described

**Taking adequate patient histories and physical examinations in resource poor settings**[10]	Physical exam/Clinical skills	3.1	Not described

**Cost-consciousness; using physical diagnosis without high technologic support**[24]	Clinical skills	3.1	Not described

^a^The percent of articles mentioning competencies was calculated by dividing the total number of articles discussing a certain competency by the total number of retrieved articles.

^b^Articles discussing competencies without examples of how these competencies were implemented were listed as not described under the category of Methods of Implementation.

Global health educational approaches were described in 18 of 32 (56.3%) identified articles [6,7,9,10,13,15-17,19,22,24,26,28-33]. The percentage of articles recommending a particular educational approach, and the suggested methods for implementing that approach, are summarized in Table 2.

**Table 2:** Educational Approaches

Educational Approach	Method of Implementation	%^a^
**Experiential Learning**		40.6
	
• **Domestic **[10,19,28,29]	• Mandatory clerkship in community medicine[10] • The first year of medical education includes a clinical and International Health and Medicine Day [28] • Community prevention outreach program at a shelter for government-assisted refugees [29] • Weekly patient contact is offered to all first-year students[19]	
	
• **International**[7,10,13,16,17,26,30-33]	• 2-week pre-clinical placement in developing countries[30,31] • Pre-departure training[10,30,31] • Two month elective at an international site[10,16,33] • Pre-departure orientations[10] • Elective terms for medical students in preclinical and clinical years[7,26] • Summer immersion experience [17] • International clinical electives, international rotations and opportunities for residents[32] • International Health Fellowship Program (IHFP) (two weeks of full-time preparatory courses followed by 6 to 8 weeks of international fieldwork)[13]	

**Didactics **[6,7,10,16,19,22,26,28,30-33]	• Student discussions [30] • Group projects[30] • Intensive courses (several weeks courses) [30,31] • Core courses[10] • Elective courses[10,30] • Meetings[10] • Global health "tracks" [31] • Student involvement in conferences [7] • Collaboration with departments of health[22] • Courses taught by activist faculty members[22] • Mandatory courses in biostatistics, epidemiology, preventative medicine and health services[26] • Introductory course [16,19,28,33] • Residency "tracks"[32] • Preclinical courses[6] • Website resources[6]	37.5

**Peer Education**[7,16,29]	• Regular global health meetings involving student leaders and senior faculty members[7] • Student-run workshops for cross-cultural communication[16] • Student leadership with family physician mentorship[29]	9.4

**Residency Training**[9,15,24]	• Global Health tracks within residency programs[9] • Mentorship[9] • Preparation for oversees electives [9] • Additional year of training outside core residency programs[9] • 4-8 weeks resident rotations[24] • Block, longitudinal didactic international health training exposure to residents[15]	9.4

**Research and Scholarly Activity**[9,32]	• Research opportunities abroad[9] • Research opportunities for residents abroad[32]	6.3

^a ^The percent of articles mentioning education approaches was calculated by dividing the total number of articles discussing a certain educational approach by the total number of retrieved articles.

The most common recommended educational approaches for teaching global health topics were didactics and experiential learning. However, there was substantial variability across described programs in the educational approach to global health as well as the methods used to implement these approaches. No commonly applied didactic method for teaching global health to medical students was apparent from this literature review. Moreover, descriptions of educational approaches often did not provide a tangible picture of what occurred in these programs.

Competencies and educational approaches were linked in 8 (25.0%) articles [7,10,17,19,24,26,28,29]. Eleven articles (34.4%) mentioned global health educational approaches or competencies for medical students, but did not provide sufficient detail to be used further [5,8,11,12,14,20,21,23,34-36]. Although these articles met search criteria, they tended to discuss international partnerships and suggestions for future endeavors rather than specifics regarding contemporary program competencies or educational approaches.

## Discussion

Interest in global health has grown dramatically among medical students in the past decade, and medical schools are grappling to define the skill sets and knowledge needed to ensure that graduates are appropriately prepared to work in this emerging field. Successful global health educational programs exist, and we explored the medical literature to identify competencies and educational approaches that might serve as potential resources for medical schools developing their own training programs. This literature review found no clear consensus on which global health competencies are relevant for most or all medical graduates to be able to draw on as future physicians. There also was little guidance regarding educational approaches for teaching global health competencies beyond the traditional methods of didactics and experiential learning.

Fifteen competencies were mentioned in the literature. The most commonly discussed ones included an understanding of the global burden of disease; travel medicine; healthcare disparities between countries; immigrant health; primary care within diverse cultural settings; and skills to better interface with different populations, cultures and healthcare systems. Although these competencies were mentioned in more than one article, no single topic area was covered in more than 16% of identified articles, suggesting a lack of consensus on the importance assigned to any particular subject. It is not possible from this review to determine why a lack of consensus exists; one possibility may be that medical schools developed their global health curricula independent of each other. Such an approach may foster innovation, but also means that the quality of resulting programs is likely to vary widely as was found in a review of global health programs [4]. Developing consensus on global health competencies would help ensure that all medical students were exposed to similar basic levels of training.

An alternative explanation for the lack of consensus in the retrieved articles is that published literature does not reflect common practice. Despite the tremendous growth in global health programs, only 11 articles were identified that addressed competencies. Furthermore, competencies were rarely the main focus of retrieved articles, giving little information on which to draw conclusions. A general consensus may exist among global health experts not reflected by the published literature.

The most common educational approaches for teaching global health were didactics and experiential learning. However, the implementation of these approaches varied considerably in the literature. Our review was hampered by the limited descriptions of educational approaches present in identified articles that may not have provided a complete picture of these programs. More detailed documentation of global health educational approaches is needed if the literature is to serve as a resource for medical schools developing new programs.

Of the 11 articles addressing competencies, 8 (72.7%) linked them to an educational approach. Conversely, educational approaches were mentioned in a majority of articles, but less than half of these linked these approaches to a competency. Competency-based descriptions of training give a more complete picture of global health education in medical curricula; schools looking to build global health educational activities should begin by defining the desired competencies, followed by enumerating the educational approaches to be used to teach them.

Over one-third of retrieved articles did not provide specific details regarding global health educational approaches or competencies despite the search strategy used. These articles often focused on the creation of international institutional partnerships to improve the quality and pace of global health curriculum development. However, establishing learning objectives and corresponding educational approaches should be prerequisites for undertaking activities such as international global health partnerships. This review highlights another potential weakness in existing global health training; medical schools may be pursuing secondary activities before establishing basic program components such as competencies and educational approaches. Without well thought-out competencies and educational approaches, medical students may lack the foundation necessary to participate in international global health programs.

Steps are underway to build consensus among global health experts regarding basic global health training for medical students. For example, the Global Health Education Consortium (GHEC) and the Association of Faculties of Medicine of Canada (AFMC) Resource Group on Global Health have created a joint committee to propose consensus global health core competencies for medical students [2]. Recently, a number of leading university-based global health programs came together to form the Consortium of Universities for Global Health (CUGH). CUGH is another potential forum for sharing global health program development information across schools. Locally, we have used the results of this review to add global burden of disease and travel-associated health topic areas to our curriculum. We suggest that medical schools use a competency based approach when developing global health programs. Educational approaches can then be linked to the learning objectives they are designed to teach. Documenting this information in the literature will facilitate the ability of medical schools to compare competencies and educational approaches being used across programs, and may stimulate consensus on appropriate global health training for medical students. Comparative studies also should be undertaken to measure how global health training affects clinical practice. Helping medical schools build appropriate global health components into their curricula should make physicians more informed and better equipped to care for patients in this increasingly globalized world.

## Conclusion

This review highlights the imperative to document global health educational competencies and approaches used in medical schools and the need to facilitate greater consensus amongst medical educators on appropriate global health training for future physicians.

## Competing interests

There are no conflicts of interest for any of the authors of the paper, including specific financial interests and relationships and affiliations relevant to the subject of the manuscript.

## Authors' contributions

RB designed the study, coordinated the research team, designed the search strategy, conducted the literature search, organized and analyzed data, and was the primary author of the manuscript. RB read and approved the final manuscript. GS conducted the literature search, organized and analyzed data, and was the secondary author of the manuscript. GS read and approved the final manuscript. NC conducted the literature search, analyzed data, and edited the manuscript. NC read and approved the final manuscript. MYC conducted the literature search and analyzed data. MYC read and approved the final manuscript. JN contributed to the design of the search strategy, conducted the literature search and analyzed data. JN read and approved the final manuscript. JH conducted the literature search, analyzed data, and edited the manuscript. JH read and approved the final manuscript. AL conducted the literature search and analyzed data. AL read and approved the final manuscript. NF conducted the literature search and analyzed data. NF read and approved the final manuscript. TB conceived and designed the study, oversaw data analysis, secured funding, and edited the final manuscript. TB read and approved the final manuscript.

## Pre-publication history

The pre-publication history for this paper can be accessed here:

http://www.biomedcentral.com/1472-6920/10/94/prepub
